# Detection of Egg Albumin in Urine

**Published:** 1917-01

**Authors:** 


**Detection of Egg Albumin in Urine.** A. Hollande, quoted in Medico-Legal Jour., July, refers to the use of egg albumin to simulate albuminuria by malingerers. (We heard of a case sometime ago in which albumin was added to urine because a person regretted an application for insurance and wished to be rejected. On the other hand, we once made an examination which was normal except for alkalinity of the urine, which is very uncommon. In this instance, the urine had been passed in anticipation of the visit. A second sample, supplied in our presence, was acid but contained albumin and casts. Probably the applicant had used the heat and nitric acid test and had over-neutralized the urine). In testing the egg albumin, either of two reagents may be used, Maurel's consists of 33% caustic soda, 25 c.c., 3% cupric sulphate, 5 c.c., glacial acetic acid up to 100 c.c.; or a mixture of equal parts of commercial formol solution and glacial acetic acid. 5 c.c. of the reagent is introduced by a pipette at the bottom of a tube containing 5 c.c. of the suspected urine. Immediately or in a few minutes, a ring precipitate occurs which increases on mixing. This does not occur with serum albumin. Rarely a ring occurs with urines containing a large amount of aceto-soluble albumin, albumoses or blood but this precipitate dissolves or, at least, does not increase when the urine and reagent are mixed. (The test might also be employed to detect egg albumin eliminated without assimilation, after raw eggs are taken.)

